# Shi's Daoyin Therapy for Neck Pain: A Randomized Controlled Trial

**DOI:** 10.1155/2018/4983891

**Published:** 2018-12-12

**Authors:** Huihao Wang, Enyu Jiang, Kuan Wang, Zhen Deng, Hongsheng Zhan, Zhibi Shen, Wenxin Niu

**Affiliations:** ^1^Shi's Center of Orthopedics and Traumatology, Shuguang Hospital Affiliated to Shanghai University of TCM, Shanghai 201203, China; ^2^Institute of Traumatology, Shanghai Academy of TCM, Shanghai 201203, China; ^3^Shanghai Yangzhi Rehabilitation Hospital (Shanghai Sunshine Rehabilitation Centre), Tongji University School of Medicine, Shanghai 201619, China; ^4^Department of Rehabilitation Engineering, Tongji University School of Medicine, Shanghai 200092, China

## Abstract

**Objective:**

To compare the immediate and short term effectiveness of Shi's Daoyin therapy (DT) rather than the Melbourne Protocol (MP) in terms of pain, mobility, and isometric strength of cervical muscles in nonacute nonspecific neck pain patients.

**Material and Methods:**

A total of 114 nonacute nonspecific neck pain patients aged 20~50 years were recruited and randomly assigned to be treated by either Shi's DT or the MP. 56 cases and 54 cases received treatment for 3 weeks and were evaluated before and after intervention and at 3-week follow-up in Shi's DT group and MP group, respectively. The outcome measures were Chinese version of the Neck Disability Index (NDI), cervical range of motion (ROM), maximal voluntary isometric force (MVIF), and pain intensity (Numeric Pain Rating Scale, NPRS).

**Results:**

All outcomes of both groups showed statistically significant improvements after the intervention and at 3-week follow-up (*P *< 0.05), while no statistically significant difference was found in NDI between groups. When followed up after 3 weeks, the ROM in axial rotation was significantly greater in the Shi's DT group (*P* < 0.05), and the NPRS in the Shi's DT group was significantly lower than the MP group (P < 0.05). At the end of the treatment period, the MVIF in lateral bending in the Shi's DT group had a lower value (*P* = 0.044) than in the MP group, but there was no significant difference in flexion and extension between the two groups.

**Conclusions:**

Both Shi's DT and MP groups demonstrated an obvious reduction in pain intensity and improvements in neck mobility after a short term follow-up period. The improvement of Shi's DT in disability and pain during functional activities is generally similar to that of the MP for the treatment of nonacute nonspecific neck pain.

## 1. Introduction

The incidence of nonspecific neck pain and its associated disorders has been measurably increasing in recent years, and nonspecific neck pain had been recognized as one of the main sources of disabilities in the general population [1]. As a serious personal, social, and economic health problem, neck pain and its associated disorders affect up to two-thirds of adults at some points in their lives [2]. It has been shown that physical exercise and training can deliver significant relief for chronic nonspecific neck and back pain, which can not only improve local symptoms but also indirectly regulate the functional status of the whole body [3–5].

The Multi-Cervical Unit (MCU, BTE Technologies, Inc., Hanover, MD) is a medical device designed for neck exercises based on the Melbourne Protocol (MP) [6]. This device showed effects in alleviating cervical dysfunction and enhancing muscle strength [7]. It could also be used to quantitatively measure neck muscle strength and cervical range of motion (ROM) [8–10]. However, such supervised rehabilitative exercise often comes at a significant expense and cannot compete with the convenience of exercise at home [11]. These devices are also difficult to popularize among nonacute nonspecific neck pain patients, especially in developing countries.

Daoyin therapy (DT) is a traditional Chinese exercise based on the principles of traditional Chinese medicine. It is characterized as an active exercise through physical action and mental coordination and has been demonstrated as effective in promoting self-care and self-treatment for physical and psychological health [12]. Shi's DT adopts head, neck, shoulder, and upper limb movements from Baduanjin and is combined with the orient and occident rehabilitation training and exercises. However, no randomized controlled studies have been performed to date to validate its therapeutic effect [13–15].

Therefore, this study intended to qualify the immediate and short term effectiveness of Shi's DT in comparison to the MP in terms of pain, mobility, and isometric strength of cervical muscles. It was hypothesized that the Shi's DT would be equally beneficial to patients with nonacute nonspecific neck pain as the MP.

## 2. Material and Methods

### 2.1. Patient Involvement

Patients were recruited from the outpatient clinic of the Shuguang Hospital Affiliated to Shanghai University of TCM from December 2015 to December 2016. The subject was diagnosed with nonacute nonspecific neck pain or related disorders by a senior clinician according to the criteria recommended by Guzman et al. [16].

Inclusion criteria for individual participation were age 18 to 50 years; nonacute nonspecific neck pain of any severity (cervical region from superior nuchal line to spine of scapula and superior border of the clavicle) with or without radiation to the shoulder region [16], with or without headaches; neck pain as main complaint for more than four weeks and no longer than one year (inclusion of patients without spontaneous recovery within four weeks) and Numerical Pain Rating Scale (NPRS) ≤ 6 points, receiving no other treatment at the same time; and provocation or reproduction of pain by neck movement or neck and head posture.

Exclusion criteria were the presence of red flags or contraindications, i.e., myelopathy or previous surgery on the cervical spine [17]; neck pain with a radicular pain pattern; entrapment neuropathy; pregnancy; whiplash injury (as the cause of the complaint); any physical treatment for neck pain in the previous three months.

All subjects gave written informed consent and were then randomly allocated to either the Shi's DT group or the MP group. Patients' priorities, experience, and preferences were not considered in this study, since both treatments were exercise therapies and subjects were randomly allocated to the two groups. The study was approved by the China Ethics Committee of Registering Clinical Trials' Ethical Review Board (No. ChiECRCT-20150040). All data including subject demographics and outcome measures were input into computer and shared among the researchers in this study. The subjects were given oral explanation of the measures but no digital or paper reports, because knowing the values of these measures is of no help to the rehabilitation process of nonacute nonspecific neck pain and may lead to unnecessary worrying. Both interventions were freely provided to the subjects, thus no burden of the intervention should be considered by the subjects.

### 2.2. Outcome Measures

Wu et al. [18] presented a Chinese version of the Neck Disability Index (NDI) specifically for Chinese-speaking individuals with neck pain. That index uses a 10-item scale to measure pain and disability felt during functional activities. It also uses a 6-point Likert scale that ranges from 0 (no disability) to 5 (complete disability) for each item. Disability ratings are assigned as follows: 0 to 4, no disability; 5 to 14, mild disability; 15 to 24, moderate disability; 25 to 34, severe disability; and above 34, complete disability. Subjects in this study were asked to complete the questionnaire at baseline, immediately after treatment, and 3 weeks after treatment.

Secondary outcome measures include pain intensity, cervical ROM, and Maximal voluntary isometric force (MVIF). Pain intensity was rated by subjects on the 11-point NPRS, for which a 0 score means no pain while 10 means the worst pain. MVIF of the cervical spine musculature was measured using MCU in 4 directions: flexion, extension, and left and right lateral bending. In addition, measures were also taken to closely observe and record any adverse reactions that occurred during the trials.

### 2.3. Sample Size Calculation

The Chinese version NDI developed by Wu et al. [18] was the primary outcome measure in the current study. A previous study reported that approximately 56% of subjects appeared to respond to MCU with NDI item scores, while the DT exercise was no less than 46% [19]. According to the noninferiority clinical trial method, with the power of 0.8, an alpha level of 0.05, and the fall rate of the case 10%, it was estimated that 55 subjects would be required for each group.

The sample size was calculated using the following equation [20]:

(1) $$\begin{array}{c} n_{T}=n_{C}=\frac{{\left(u_{\alpha }+u_{\beta }\right)}^{2}\left[\pi_{T}\left(1-\pi_{T}\right)+\pi_{C}\left(1-\pi_{C}\right)\right]}{{\left[\left(\pi_{T}-\pi_{C}\right)-\delta \right]}^{2}} \end{array}$$

were *n*_*T*_ and *n*_*C*_ denoted the sample sizes of the two groups, respectively. *u*_*α*_ + *u*_*β*_ were the cumulative distribution function of a standard normal deviate, based on the *α* (type I error) and *β* (type II error). *π*_*T*_ and *π*_*C*_ denoted the success proportion with MP and DT, respectively. *δ* was the testing margin.

### 2.4. Randomization and Allocation

The clinician who made the diagnosis performed the subject enrollment and intervention assignment. After suitable subjects were filtered by the acceptance criteria, their basic information would be transmitted to a designated person in random allocation center (Statistical Division), who was not involved in the rest of the study. The subjects were randomly assigned to the Shi's DT group or the MP group in a 1:1 ratio by using random numbers generated by computer.

### 2.5. Interventions

#### 2.5.1. Shi's DT Group

**Figure 1:**
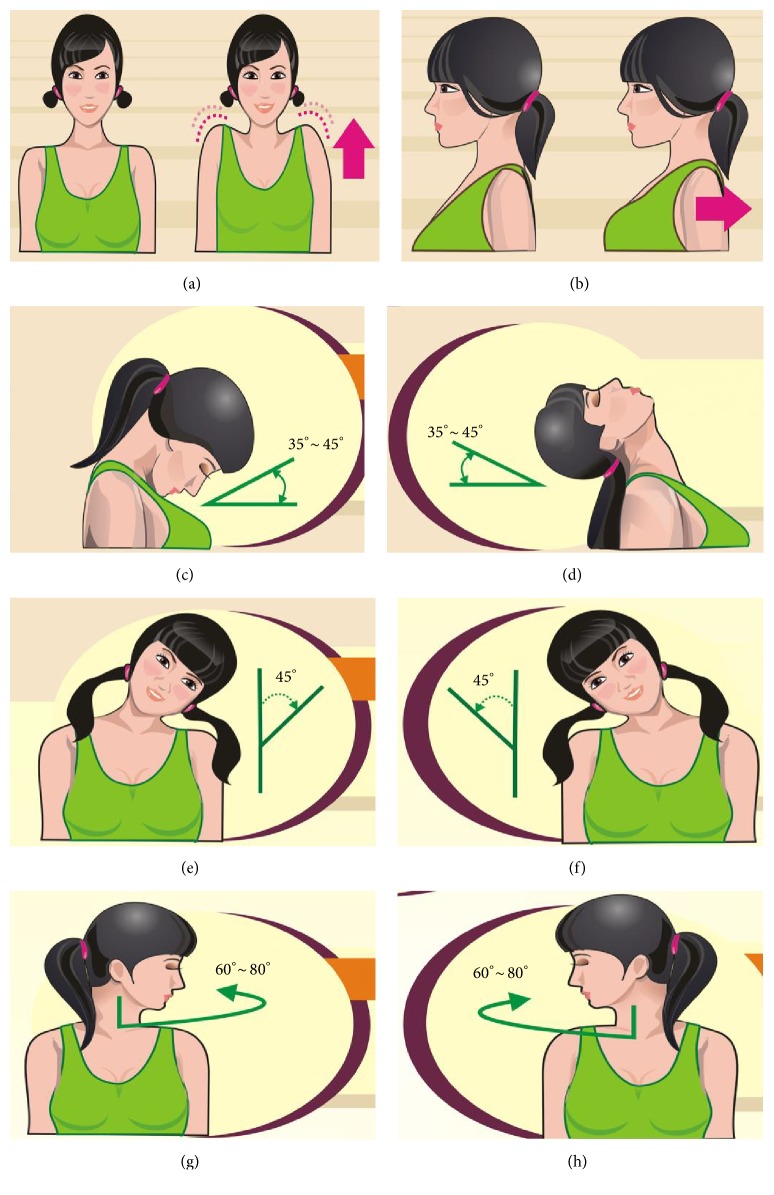
Procedure of Shi's Daoyin therapy. ((a) Upward movement of the shoulders; (b) backward movement of the shoulders; (c) cervical flexion between 35°~45°; (d) cervical extension between 35°~45°; (e) cervical left lateral bending about 45°; (f) cervical right lateral bending about 45°; (g) cervical left rotation between 60°~80°; and (h) cervical right rotation between 60°~80°).

Subjects allocated to the Shi's DT group were required to stretch the neck, shoulder, and upper limbs for 5 minutes under the guidance of the same rehabilitation therapist and then were performed through the following procedure in Figure 1.

*Start Gesture (Figure 1(a))*. Two feet stand straight apart with the shoulders width. Let the arms and hands hang down loosely and maintain gentle, natural breathing. The next step will start while subjects focus on the breathing gently.

*Shoulder Movement (Figure 1(b))*. This step aims to relax the tendons by moving the shoulders backward and bringing the back into an erect position. The shoulders are then rotated 10 times in a clockwise motion. The muscles need to keep tense during the upward and backward motion and to relax while rotating downwards. The motion is then repeated in an anticlockwise direction. All actions need to be coherent, slow, and uniform.

*Cervical Spine Movement (Figures 1(c)–1(h))*. This movement aims to eliminate muscle tension by exercising the cervical spine in 6 directions, through flexion, extension, left and right lateral bending, and left and right rotation. The head/neck is rotated to near-physiological limit for each movement and maintains in this position for 3 seconds, before returning to the neutral position. Each movement is repeated 2~4 times. After moving head through all 6 directions, the subject will have completed one set. Subject is permitted to attempt 3~4 sets if their condition allowed and is required to practice 5~8 minutes per time, at 3 times per day.

*Notes for Shi's DT Group*. (1) Subjects with acute neck pain are not suitable for practicing Shi's DT. (2) Each neck movement should reach the near-physiological limit, which requires slow movement and coordinated breathing. (3) The subject should immediately stop exercising and turn to a physician if he/she appears to develop aggravating symptoms of neck pain, dizziness, nausea, or other related symptoms.

#### 2.5.2. The MP Group

Subjects allocated to the MP group were required to stretch the neck, shoulder, and upper limbs for 5 minutes under the guidance of the same rehabilitation therapist and complete the preliminary test. The individualized training plan will be developed by the MCU. Then, subjects completed all exercises under the guidance of the same rehabilitation therapist according to the MP based on MCU.

Subjects sat on the chair of the MCU and were secured by belts and straps. The height and direction of the chair and the back were adjusted to fit the height and position of the subject. The Halo frame was locked in position and the subject was helped to enter the following information by the rehabilitative therapist before beginning the training program:

1. Log into the training interface.
2. Click the training program for cervical flexion, extension, left lateral bending, right lateral bending, left rotation, and right rotation.
3. Choose the groups and times per group for the subject according the individualized training plan.
4. Choose the weight of the subject according the individualized training plan.
5. Choose the interval between breaks according the individualized training plan. Subjects were trained twice a week for 30~ 40 minutes each time.

**Figure 2:**
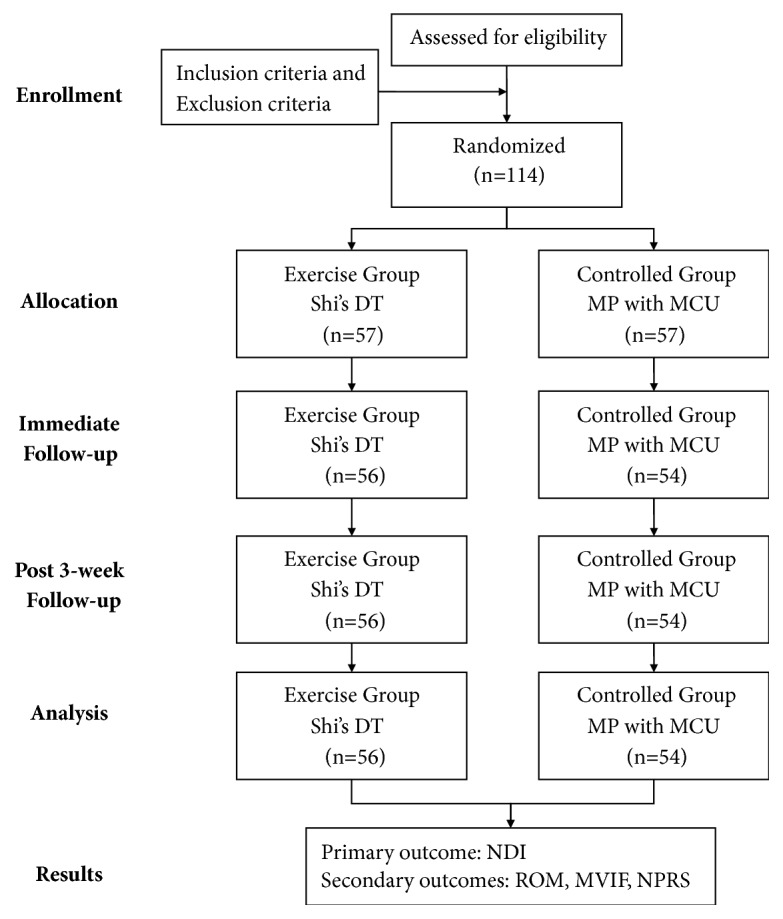
Flowchart of subjects and follow-up evaluation. (NDI, Neck Disability Index; ROM, cervical range of motion; MVIF, maximal voluntary isometric force; NPRS, Numeric Pain Rating Scale).

The treatment course for both the DT and MP groups was 3 weeks. The outcomes would be evaluated after the intervention and at 3-week follow-up (Figure 2). All data was recorded and statistically analyzed by the same therapist.

### 2.6. Data Analysis

Case data was processed and analyzed by the Statistical Package for Social Sciences version 21.0 for Windows (SPSS, Inc., Chicago, IL, USA). The statistical description of data that did not conform to a normal distribution was described by M (Q1, Q3). A* T *test or rank sum test was used to compare the pre- and posttest data. A group* t* test or rank sum test was used to compare between groups. Count data used the* x*^2^ test, while general statistical tests were performed with two-sided tests.* P*<0.05 indicated that the difference was statistically significant. The analysis was planned to be an intention-to-treat analysis involving all subjects who were randomly assigned.

This study adopted the no inferiority test, with *α* = 0.05 for a unilateral test.* B *= 0.20, if* P *≤ 0.05, indicated that H0 was refused, meaning the effect of Shi's DT was not inferior to the MP; if* P *> 0.05, then the no inferiority conclusion could not be made.

## 3. Results

### 3.1. Patient Demographics

A total of 114 patients were eligible for this study and were randomly allocated to either Shi's DT group or MP group. Fifty-six subjects in the Shi's DT group finished the session and 1 case dropped out (declined to finish the treatment and be followed up) during the treatment period, while 54 subjects in the MP group finished the session and 3 cases dropped out (declined to finish the treatment and be followed up). Totally, 110 subjects finished the 3-week intervention and 3-week follow-up. There was no significant difference at demographic baseline between groups (Table 1).

### 3.2. Main Outcomes

The NDI significantly improved (*P *< 0.05) within each of the two groups immediately after the intervention and at 3-week follow-up. However, there was no significant difference in NDI between the two groups at the end of the exercise program. When followed up after 3 weeks, the NDI of two groups still showed no significant difference (Table 2).

### 3.3. Secondary Outcomes

**Figure 3:**
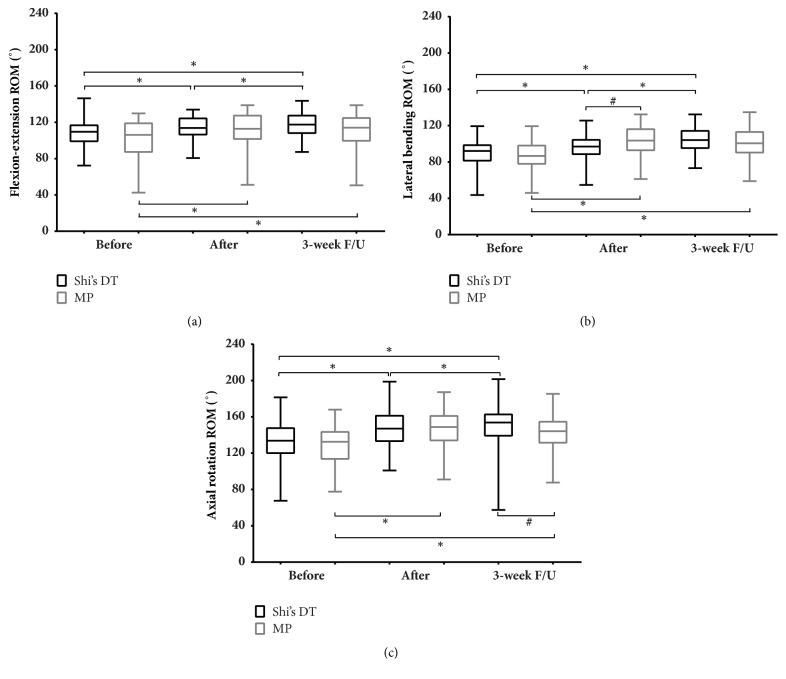
Comparison of neck range of motion (ROM) in (a) flexion-extension; (b) lateral bending; and (c) axial rotation between and within groups. Note: the data were used in a K-S test for statistical analysis. Observation time points: (1) before intervention; (2) after intervention; and (3) 3-week follow-up (F/U). *∗*,* P* < 0.05 with Wilcoxon signed rank test compared with data from 3 points in the same group; #,* P* < 0.05 with Mann–Whitney U test compared between groups at the same time.

At the end of the exercise program, there was a significant difference between the two groups in the ROM of the neck in lateral bending (*P *< 0.05). The ROM in the other directions did not show any significant difference. When followed up after 3 weeks, the ROM in the Shi's DT group further increased in all directions (*P *< 0.05), and the ROM in axial rotation was significantly greater in the Shi's DT group (*P *< 0.05) than the MP group. The ROM in the other directions did not show any significant difference between the two groups at 3-week follow-up (Figure 3).

**Figure 4:**
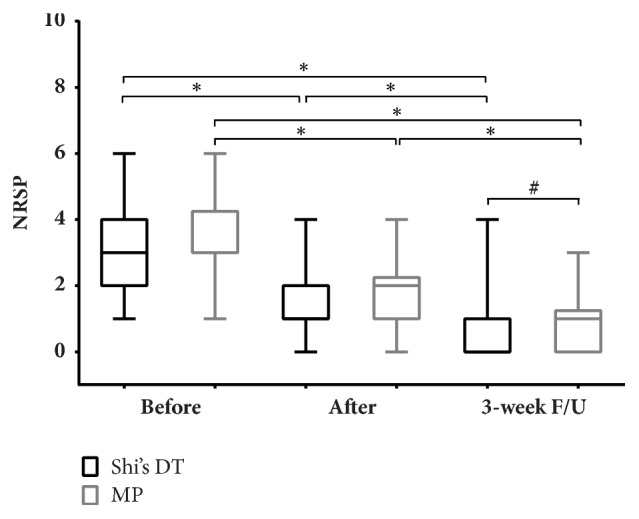
Comparison of numerical pain rating scale (NPRS) between and within groups. Note: the data were used in a K-S test for statistical analysis. Observation time points: (1) before intervention; (2) after intervention; and (3) 3-week follow-up (F/U). *∗*,* P* < 0.05 with Wilcoxon signed rank test compared with data from 3 points in the same group; #,* P* < 0.05 with Mann–Whitney U test compared between groups at the same time.

There was a significant difference in the NPRS score before and after exercise for both groups (*P *< 0.05). Also, at the end of the exercise session, the NPRS score was not significantly different (*P* > 0.05) between the groups. However, when followed up after 3 weeks, the NPRS in the Shi's DT group showed a significantly lower value than the MP group (*P* < 0.05). For both groups there was a significant improvement in NPRS from before treatment to 3-week follow-up (*P* < 0.05) (Figure 4).

**Figure 5:**
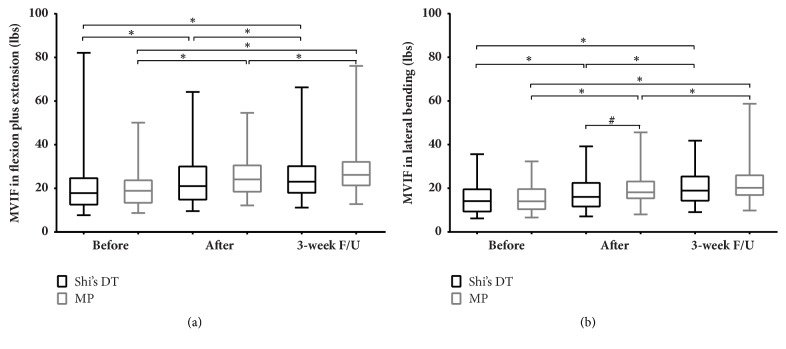
Comparison of maximal voluntary isometric force (MVIF) in (a) flexion plus extension and (b) lateral bending between and within groups. Note: the data were used in a K-S test for statistical analysis. Observation time points: (1) before intervention; (2) after intervention; and (3) 3-week follow-up (F/U). *∗*,* P* < 0.05 with Wilcoxon signed rank test compared with data from 3 points in the same group; #,* P* < 0.05 with Mann–Whitney U test compared between groups at the same time.

At the end of the treatment period, the MVIF in lateral bending in the Shi's DT group had a lower value (*P *= 0.044) than in the MP group, but there was no significant difference in flexion and extension between the two groups. When followed up after 3 weeks, the MVIF in flexion and extension showed no significant difference between the two groups (Figure 5).

No adverse event was noticed during the treatment period or after 3-week follow-up in either group.

## 4. Discussion

This study compared the immediate and short term effectiveness of Shi's DT to the MP in terms of pain intensity, mobility, and isometric strength of cervical muscles in patients with nonacute nonspecific neck pain. The results showed that all outcomes of both groups significantly improved within the groups after the intervention and at 3-week follow-up. The Shi's DT had some advantages in improving NRSP and ROM in axial rotation, while the MP showed some advantages in improving ROM and MVIF in lateral bending. It was demonstrated that the effect of Shi's DT is comparable to that of MP in treating nonacute nonspecific neck pain.

The normal balance of the cervical spine is dynamic and very important for maintaining the stability of the neck, including vertebral bodies, attachments, intervertebral discs, ligaments, and neck. Interfering with this balance can lead to cervical spondylosis, inducing neck pain, and relative disorders [21]. Studies have confirmed that cervical muscle strength and ROM in patients with cervical spondylosis is significantly decreased and the musculature around the cervical spine is relatively easier to fatigue [6, 8, 10]. Active exercise intervention is a key method for correcting dynamic unbalance of the cervical spine [22, 23]. However, subsequent radiologic examinations often do not produce positive results, which can be frustrating for both patients and physicians. The MCU device was developed to detect and evaluate the patient's cervical function with the actual dynamic physiological state, thus providing prognostic evaluation and a plan for follow-up treatment.

The most distinguishing characteristic between DT and modern physical exercise is that the former pays more attention to the control of the rhythm of breathing and meditation [15]. Shi's DT was developed through selecting movements from Baduanjin and combining with the orient and occident rehabilitation training and exercises. One particular movement requires lifting, abduction, suppression, and adduction of the scapula with the aim of exercising the trapezius, the scapula, the rhombus, and the serratus anterior muscles. The trapezius and the levator scapula belong to the upper limb muscles and act to maintain the stability of the cervical spine. In addition, disorders of the rhomboid and anterior sawing muscles could evoke pain in the junction between the neck and chest and also lead to shoulder pain. Training these muscles, in addition to rhythmic breath and meditation, may help to relieve episodes of spasmodic and tense muscles. DT aims to ease muscle tension in the cervical spine by exercising the neck through flexion and extension, left and right flexion and left and right rotation. By regularly practicing DT, the cervical muscle group can be fully drawn, loosening the adhesion of cervical facets and surrounding soft tissues, reducing muscle tension, enhancing muscle strength, restoring exogenous and endogenous stability of cervical spine, and establishing a new dynamic balance [24–26].

In this study, the NDI score and ROM were taken as the main outcome measures, because both of these could reflect the subjective and objective condition of the patient from two aspects: the degree of nonspecific neck pain and the influence of related symptoms on daily life. Improvements in cervical spine dysfunction were roughly the same. This result preliminarily demonstrated that Shi's DT had the same short term efficacy as MP in improving symptoms of nonacute nonspecific neck pain.

In terms of objective indicators, at 3-week follow-up subjects treated with Shi's DT demonstrated a greater ROM than the MP group. One likely reason is that the Shi's DT group was required to slowly exert effort to reach the physiological limit in each movement. Through regular training, up to 3 times per day for 3 weeks, patients were able to fully stretch the neck muscles, reduce muscle tension, and release the cervical facet joints to increase the neck mobility. This study had a 3-week follow-up period, but using Shi's DT in this way may easily be extended to routine daily life.

Regarding muscle strength, the MP group demonstrated significantly greater lateral bending strength over the Shi's DT group. This is likely due to the use of resistance equipment when treating with MP. Repeated resistance to force could stimulate muscle fibers and increase the strength of neck muscles.

To summarize, clinical improvements observed with the use of Shi's DT were comparable to using MP with MCU for nonacute nonspecific neck pain. Major advantages of Shi's DT are that it is easier to practice without assistance and the treatment will not suffer from space limitations. Shi's DT may also be incorporated into daily practice after the treatment plan has finished to help prevent the recurrence of nonspecific neck pain. It is most suitable for patients with moderate to mild nonspecific neck pain.

There were also some limitations in this study. Firstly, we used a short follow-up period for both groups. Since the questionnaires about quality of life such as short form-36 health survey questionnaire (SF-36) are always applied in a long period of time, we did not use them in the current study. Secondly, due to lack of long-term follow-up, it could not be concluded whether Shi's DT could significantly reduce the long-term recurrence rate. Future research should consider a longer-term study to investigate the effect of Shi's DT on the nonacute nonspecific neck pain.

## 5. Conclusions

Both Shi's DT and MP groups demonstrated an obvious reduction in nonacute nonspecific neck pain and improvements in neck mobility after a short term follow-up period. The improvement of Shi's DT in disability and pain during functional activities is generally similar to that of the MP for the treatment of nonacute nonspecific neck pain.

## Figures and Tables

**Table 1:** Baseline characteristics of subjects.

	**Shi's DT group**	**MP group**	***P* value**
**Number**	57	57	
**Age (year)** Mean (SD)	32.79 (7.53)	34.40 (7.09)	0.241
**Gender (n)**			
Female	30	33	0.572
Male	27	24	
**Duration**			
≤3 months	22	19	0.840
3~6 months	8	9	
6~12 months	27	29	
**NDI (0-50)**			
M (Q1, Q3)	12(8, 20)	13(6, 20.5)	0.744
**ROM (**°**)**			
Flexion and extension			
M (Q1, Q3)	109.60 (99.10, 116.70)	106.15 (87.34, 118.95)	0.308
Left and right bending	92.19	86.68	
M (Q1, Q3)	(81.47, 98.55)	(77.95, 98.12)	0.217
Left and right rotation	133.70	132.49	
M(Q1, Q3)	(112.00, 147.52)	(113.72, 143.42)	0.371
**NPRS**			
(0-10) M(Q1, Q3)	3 (2, 4)	3 (3, 4.25)	0.144
**MVIF**			
Flexion and extension			
M (Q1, Q3)	17.85 (12.58, 24.68)	18.90 (13.40, 23.68)	0.520
Left and right bending			
M (Q1, Q3)	14.10 (9.35, 19.50)	14.00 (10.40, 19.60)	0.503

Notes: Data that did not conform to a normal distribution was described by M (Q1, Q3). Shi's DT group, Shi's Daoyin therapy group; MP group, Melbourne Protocol (MP) with the Multi-Cervical Unit (MCU); NDI, Neck Disability Index; ROM, range of motion; NPRS, Numerical Pain Rating Scale; MVIF, maximal voluntary isometric force.

**Table 2:** Comparison of NDI between and within groups *M*(*Q*_1_, *Q*_3_).

group	Before exercise (%)	After exercise (%)	3-week follow-up (%)
Shi's DT	12(8,20)	6(3.25,12)^△^	2(0,4)^△,*∗*^
MP	13(6,20.5)	6(2,10)^△^	4(2,8)^△,*∗*^

*Z*	-0.327	-0.922	-1.742
*P*	0.744	0.357	0.082

Note: The data was used in a K-S test for statistical analysis. Observation time points: (1) before intervention; (2) after intervention; and (3) 3-week follow-up. △, P < 0.05 with Wilcoxon signed rank test compared with data before exercise in the same group. *∗*, P < 0.05 with Wilcoxon signed rank test compared with data after exercise in the same group.

## Data Availability

The data used to support the findings of this study are available from the corresponding author upon request.
